# Factori care influențează biopsia ureteroscopică la pacienții cu tumori de cale urinară superioară

**Published:** 2014

**Authors:** Niță Gh, Mulțescu R, Drăguțescu M, Arabagiu I, Persu C, Geavlete P

**Affiliations:** * Spitalul Clinic de Urgență “Sf. Ioan”, București, România

**Rezumat**

**Introducere.** Tumorile uroteliale de cale urinară superioară (TUCUS) “low grade” pot beneficia de tratament conservator. Stadializarea preoperatorie corectă este esențială. Scopul acestei lucrări este analiza retrospectivă a corelațiilor între examenul histopatologic după biopsia ureteroscopică și cel obținut după nefroureterectomie la pacienții cu TUCUS.

**Material și metodă.** In perioada 1998 - 2013 în cadrul Clinicii de Urologie a Spitalului Clinic de Urgență “Sf. Ioan” București au fost diagnosticați și tratați pentru TUCUS un număr de 195 pacienți. Pentru biopsie s-au utilizat pense de 5 sau 3 F, sonde cu coșuleț, sau ureterorezectoscopul Storz. Dintre cele 98 cazuri la care s-a practicat biopsie ureteroscopică, s-a indicat nefroureterectomie radicală la 38 cazuri. S-a analizat retrospectiv, la 35 cazuri, corelația între rezultatul histo-patologic al fragmentelor tumorale obținute prin biopsia ureteroscopică și cel final al piesei operatorii (după nefroureterectomie).

**Rezultate.** Dintre cele 35 cazuri analizate, biopsia ureteroscopică a stabilit diagnosticul de tumoră urotelială în 32 de cazuri (91.4%). La 2 cazuri fragmentele au fost de foarte mici dimensiuni și nu au putut fi utilizate pentru diagnostic, iar la un caz s-a diagnosticat o leziune benignă. La cele 32 de cazuri cu diagnostic ureteroscopic de tumoră urotelială s-a stabilit o corelație între grading-ul după biopsie și cel după nefroureterectomie la 84,37% din pacienți (27 cazuri). Ceilalți 5 pacienți au fost subevaluați preoperator.

**Concluzii.** Biopsia ureteroscopică a TUCUS nu permite stadializarea tumorală cu acuratețe datorită fragmentelor de mici dimensiuni care nu includ toate straturile segmentului afectat. Totuși, diagnosticul ureteroscopic se corelează semnificativ cu examenul histopatologic în ceea ce privește stabilirea gradingului tumoral, cu rol esențial în stabilirea unei atitudini terapeutice, mai ales în cazurile la care se urmărește un tratament conservator. 

**Cuvinte-cheie:** biopsie ureteroscopică, calea urinară superioară, TUCUS

